# Carbohydrates Used in Polymeric Systems for Drug Delivery: From Structures to Applications

**DOI:** 10.3390/pharmaceutics14040739

**Published:** 2022-03-29

**Authors:** Xiangjie Di, Xiao Liang, Chao Shen, Yuwen Pei, Bin Wu, Zhiyao He

**Affiliations:** 1 Department of Pharmacy, State Key Laboratory of Biotherapy and Cancer Center, National Clinical Research Center for Geriatrics, West China Hospital, Sichuan University, Chengdu 610041, China; dixiangjie17@mails.ucas.ac.cn (X.D.); xiaoliang9101@163.com (X.L.); s545008001@163.com (C.S.); peiyuwenkaoyan@163.com (Y.P.); binw83@hotmail.com (B.W.); 2 Clinical Trial Center/NMPA Key Laboratory for Clinical Research and Evaluation of Innovative Drug, West China Hospital, Sichuan University, Chengdu 610041, China; 3 Department of Gynecology and Obstetrics, West China Second Hospital, Sichuan University, Chengdu 610041, China; 4 Key Laboratory of Drug-Targeting and Drug Delivery System of the Education Ministry, Sichuan Engineering Laboratory for Plant-Sourced Drug and Sichuan Research Center for Drug Precision Industrial Technology, West China School of Pharmacy, Sichuan University, Chengdu 610041, China

**Keywords:** carbohydrate-based polymer, drug delivery, hydrogel, nanoparticle

## Abstract

Carbohydrates, one of the most important compounds in living organisms, perform numerous roles, including those associated with the extracellular matrix, energy-related compounds, and information. Of these, polymeric carbohydrates are a class of substance with a long history in drug delivery that have attracted more attention in recent years. Because polymeric carbohydrates have the advantages of nontoxicity, biocompatibility, and biodegradability, they can be used in drug targeting, sustained drug release, immune antigens and adjuvants. In this review, various carbohydrate-based or carbohydrate-modified drug delivery systems and their applications in disease therapy have been surveyed. Specifically, this review focuses on the fundamental understanding of carbohydrate-based drug delivery systems, strategies for application, and the evaluation of biological activity. Future perspectives, including opportunities and challenges in this field, are also discussed.

## 1. Introduction

**Figure 1:**
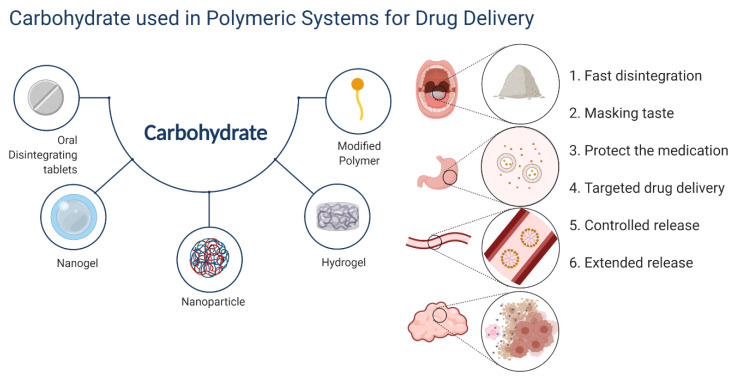
Carbohydrates used in polymeric systems for drug delivery.

A polymer is a substance composed of macromolecules whose structure is composed of multiple repeating units. Because polymers contain a variety of functional groups, they can be blended with other high- and low-molecular-weight materials to impart unique functional properties, be conjugated with some drugs, and can be tailored for any application [1,2,3]. Similar to other polymers, carbohydrates have many active groups. The general formula of carbohydrates is C_x_(H_2_O)_y_, which has a carbonyl, an aldehyde or a ketone. When monosaccharides dissolve in solution, they exist as pyranose or furanose, and acquire an asymmetric center called “anomeric carbon”. The hydroxyl group on anomeric carbon forms a glycosidic bond for a polysaccharide or a glycan chain in the α-/β-configuration with the hydroxyl group. Simultaneously, amidogen and/or hydroxyl groups at other sites can be further modified to obtain desirable physical and chemical properties [4,5]. As natural macromolecules and biologically active substances, as shown in Figure 1, carbohydrates play a very important role in polymeric drug delivery systems.

This review will focus on carbohydrate-based and carbohydrate-modified polymeric systems for drug delivery. It will specifically focus on the preparation of targeted polymers, the synthetic method for modifying polymers by carbohydrates, and applying the materials in drug delivery systems.

## 2. Carbohydrates Used as Key Excipients for Novel Drug Delivery Systems

### 2.1. Carbohydrates Used in Orally Disintegrating Tablets (ODTs)

Oral drug delivery is the safest, most convenient, and most economical method of drug delivery with high patient compliance. However, there are several problems with this kind of pharmaceutical, such as choking and swelling, which are difficult in geriatric and pediatric patients, and sometimes the acute onset of disease. ODTs are a novel drug delivery system that can solve these problems [6]. As an excipient, carbohydrates play a very important role in the distribution of this pharmaceutical. 

Starch is a cheap and renewable material, and has the ability to form tasteless, odorless, and biodegradable tablets [7]. Moreover, after starch is modified, this material can form a rapidly dissolving tablet. In 2018, Garcia et al. used pregelatinized starch as an excipient to form ODTs to improve a huge increase in hydrophilicity, provide a fast disintegration time and establish a higher loading efficiency compared to other tablet delivery systems [8]. Cyclodextrins (CDs), as cyclic carbohydrates, are produced from starch or its derivatives using CD glycosyltransferase. This material was first separated and purified by Antoine Villiers in 1891, and because they can form noncovalently bonded inclusion complexes with many compounds, CDs have been applied as pharmaceutical excipients [9]. As a classic antipsychotic drug, clozapine is often used to treat schizophrenia, regardless of positive and negative symptoms. It can also be used to inhibit mania and neuroleptic responses. However, because of its characteristics, such as good lipid solubility and low aqueous solubility, the bioavailability of clozapine is limited, and patient compliance is not very good. In 2013, Zeng et al. prepared a Clozapine tablet that included hydroxypropyl-β-CD by an evaporation method. This kind of dosage form has a faster absorption rate and higher absorption than other clozapine dosage forms because hydroxypropyl-β-CD can improve inclusion complexing ability, increase water solubility, and lower toxicity [10,11,12].

The application of carbohydrates in ODTs as excipients has stretched beyond the laboratory stage and been used in many commercial applications. For example, Wowtab, a patented technology developed by Yamanouchi Pharma Technologies Inc., uses low- and high-moldability carbohydrates to obtain a tablet that has an acceptable hardness and fast dissolution rate. This technology is applied to diphenhydramine, named Benadryl Fastmelt, to achieve the target of rapid release and increase children’s compliance. Other OTDs, such as Allegra, NuLev, Dolflash, Risperdal Mtab, and Proxalyoc, have also been commercialized.

### 2.2. Carbohydrates Form Inclusion Complexes with Drugs

As an excipient, carbohydrate is not only used to form a tablet but also forms inclusions with drug molecules. For example, in 2020, Topuz et al. used cyclodextrin (CD) molecules as starting materials to produce nanofibers. These nanofibers, compared with other kinds of nanofibers, can carry larger numbers of antibiotics because of cyclodextrin’s unique properties [13,14,15]. Simultaneously, the solubility of the antibiotics was increased drastically. These effects are achieved with CD’s unique toroidal shape, of a hydrophobic cavity inside and a hydrophilic cavity outside. This structure of CD molecules means that they can host organic and inorganic molecules in their cavities. After the formation of this complex is finished, the hydrophilic exterior of CD makes them more soluble. From this characterization, researchers have developed many different usages, such as working with the genetic therapeutic agents of cancer [16], improving solubility and the sustainable release of drugs [17], and targeting DNA for drugs [18].

### 2.3. Carbohydrate-Based Materials as Scaffolds in Drug Delivery Systems

Carbohydrate scaffolds have been used extensively. For example, polymers, nanoparticles, and surfaces are possible drug delivery systems [19,20,21,22,23]. The features of carbohydrates, such as biocompatibility and the of forming supramolecular networks, are very useful to the creation of hydrogel, nanoparticles, and nanogel drug delivery systems. In addition, carbohydrate-based polymers can be functionalized easily to improve solubility, stability, encapsulation, and responsiveness [24,25]. Therefore, they have been applied increasingly extensively in drug delivery.

Broadly speaking, nanoparticles are usually defined as particles of matter that are between 1 and 100 nanometers in diameter [26]. There are three significant properties of nanoparticles: they are highly mobile in the free state and have enormous surface and quantum effects. These effects allow nanoparticles to have a broad range of applications in drug delivery. Because of the nature of carbohydrates, nanoparticles made by carbohydrates have the advantages of protecting the drug from enzyme degradation, controlling release, targeting the affected area, and improving bioavailability. As nanoparticles are composed of hydrogels, nanogels have the advantages of both nanoparticles and hydrogels. They can be used as drug delivery agents, contrast agents for medical imaging, nanoactuators, and sensors [27,28,29,30,31]. We will subsequently discuss different kinds of polysaccharides in the drug delivery system.

## 3. The Structures, Characteristics and Applications of Different Carbohydrate Polymers in Drug Delivery Systems

As a natural product, polysaccharides have a very rich structure, which leads to their different characteristics, especially in drug delivery. Next, we will describe each type of polysaccharide separately and then elucidate their application in drug delivery.

### 3.1. Xanthan Gum

Xanthan gum (XG) is a high-molecular-weight carbohydrate produced by the gram-negative bacterium Xanthomonas and approved by the US FDA as a food additive in 1969 [32,33,34]. The traditional method to produce XG is shown below. First, the selected strain is expanded by growth on solid or liquid bacterial growth medium to obtain the inoculum for bioreactors that are large enough to produce. By adjusting factors such as the type of bioreactor used, the mode of operation, the medium composition, and the culture conditions, the microorganism and xanthan production can be controlled. At the end of the fermentation, bacterial cells are removed by filtration or centrifugation. After separation, precipitation, dewatering, and drying, pure XG can be obtained [35].

**Figure 2:**
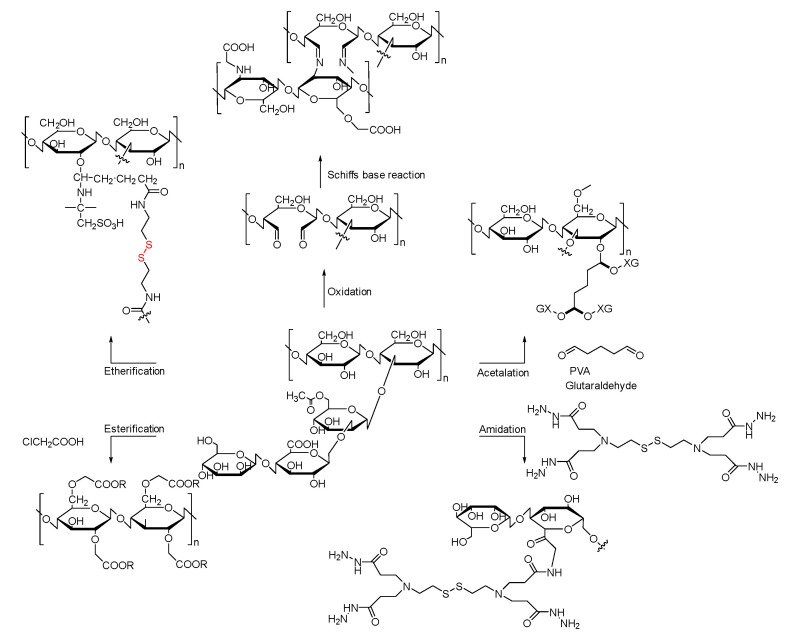
The structure and different modifications of Xanthan gum.

XG consists of a β-1,4-d-glucopyranose pillar with three units of saccharide side chains composed of mannose and glucuronic acid, as shown in Figure 2. When pH > 4.5, the acetyl and pyruvate will deprotonate, and the charge density of these XG chains will increase, allowing XG chains to crosslink to each other by ions. The glucuronic acid in the side chain makes XG chains polyanionic [36,37]. Because of its structure, XG can crosslink with many different organic or inorganic substances to form nanogels, fibers, nanoparticles, or microbeads. These materials can achieve pH-responsive, redox-responsive, and sustained/controlled drug delivery.

In the research area of XG on drug delivery systems, the most popular direction in recent years has been hydrogels and nanogels. These can be used for locally targeted sustained drug release and controlled release. Sethi et al. crosslinked XG with starch using microwaves to develop a novel acrylic acid-grafted hydrogel. This hydrogel has excellent swelling capacity and a special response to pH. They found that the swelling behavior of this XG-starch crosslinking gel depended on the pH of the environment. At room temperature, the increase in the swelling ratio depends on the increase in the pH. This result suggests that the XG–starch–hydrogel might be very useful for colon-specific drug delivery [38].

**Figure 3:**
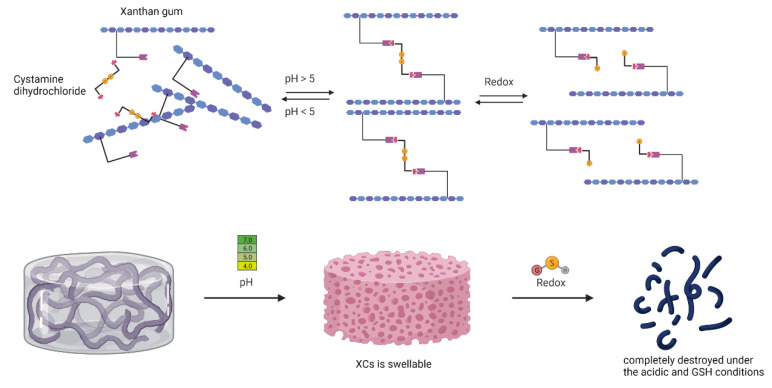
The mechanism of pH/redox-responsive delivery.

Controlled redox- or pH-responsive drug release is another application of XG. In 2019, Zhang et al. used “one-pot” synthesis to crosslink XG with cystamine tetra-hydrazide in water solution to obtain special pH/Redox-responsive materials that can form nanogels with 117–615-nm diameters. The source of this dual-responsiveness was the disulfide bonds and amino groups from cystamine tetrahydrazide and the carboxyl groups from XG in these polymers. After loading DOX in this nanogel, the researchers tested drug release at different pH values and reductive conditions. Similar to Figure 3, the results showed that with low pH and the reducing agent L-glutathione (GSH), DOX cumulative release at the equilibrium state was almost 72.1%. This number was higher when compared with the situation with only low or medium pH [39]. Therefore, modified XG can achieve different effects by using different modifying groups or crosslinking chains.

Another application of XG is embedding inorganic nanoparticles. Xanthan gum is used as a surface stabilizing agent covering the inorganic nanoparticles. This application can increase the delivery of curcumin in cancer treatment and decree biocompatibility and cytotoxicity [40,41,42]. However, because it is a form of surface modification, we will not discuss this topic here.

Xanthan gum is an attractive natural polysaccharide in terms of its high potential for biomedical applications. It offers numerous possibilities for researchers to develop new sustained-release formulations.

### 3.2. Trans

**Figure 4:**
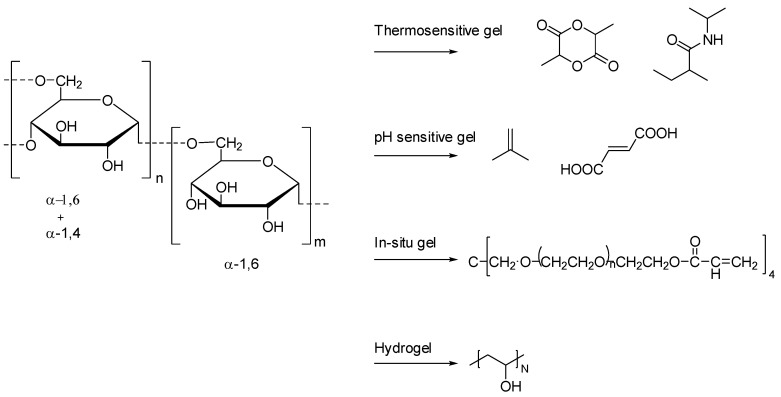
The structure of dextran and its modification in drug delivery.

Dextran is a branched polymer consisting of d-glucose; the major chain is formed by alpha C-1 to C-6 bonds, as shown in Figure 4. The molecular weight of this carbohydrate ranges from 2 to 3000 kilodaltons. As a natural polymer, dextran has several special characteristics, such as biodegradability, nonspecific adsorption of cells, resistance to protein adsorption, and ease of gain and modification [43]. Because of this characteristic, dextran is extremely popular in drug delivery systems. Although the metabolic process of dextran in vivo is not clearly understood, with further research, its application in drug delivery systems will be more widely used.

**Figure 5:**
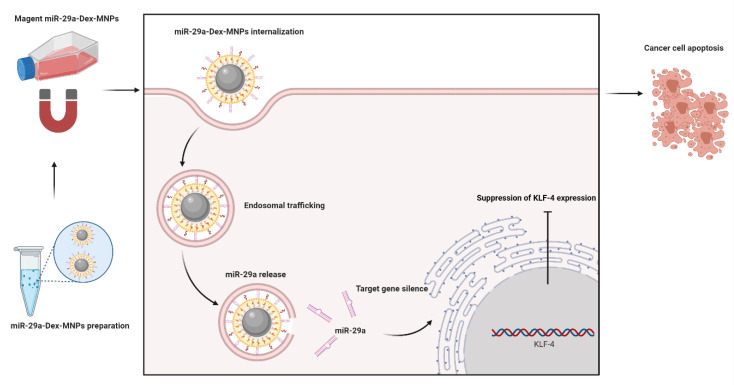
Dextran used in RNA interference therapy.

One application of dextran is the delivery of unstable or difficult drugs across cellular membranes [44]. As a novel material for drug delivery, dextran nanoparticles have many advantages, such as good biocompatibility, nontoxicity, good biodegradability, improved stability, improved bioavailability, and increased drug absorption [45]. As it can be prepared into nanoparticles by many different methods, dextran can be applied in many different types of drugs. For example, RNA interference therapy is a novel treatment method that can be used to treat cancer or genetic diseases such as Duchenne muscular dystrophy [46]. However, it has many problems; for example, unpackaged RNA is rapidly degraded in the biological environment by ribonuclease, so the drug delivery system is very important for RNA interference therapy [47]. In 2020, Pieterjan Merckx et al. used a cationic dextran-based nanogel as the core to carry siRNA, which has a high loading ability for RNA of 0.4 mg and confirmed RNA delivery potential in many different kinds of cell types. To enhance particle stability and intracellular RNA delivery at the same time, they used pulmonary surfactant (PS) as a coat on this nanogel. After this hybrid addition, the RNA-NP-PS complex could tolerate continuous lyophilization and nebulization [48]. Additionally, dextran can deliver traditional drugs. Poly(lactic acid glycolic acid) and dextran can form micelles by self-assembly of the parent graft by esterification. It can encapsulate paclitaxel to form a drug delivery system suitable for injection, avoid the disadvantages of paclitaxel, such as poor water solubility, and have many side effects, with high antitumor activity and low toxicity [49]. As a hydrogel, dextran has another usage. In 2019, Yalcin et al. used dextran-coated iron oxide nanoparticles to deliver miR-29a to tumors to inhibit the expression level of tumor miRNAs. Although miRNA has a tiny half-life and is very unstable, they established a platform that could protect miR-29a by dextran-coated magnetic nanoparticles for cancer therapy. According to this study’s results, they showed that dextran-coated magnetic nanoparticle formulation is a safe delivery system, and the usage of magnetic nanoparticles can protect the unstable miR-29a to avoid decomposition and carry this miRNA to the inside of cancer cells, as we describe in Figure 5. These results suggest that dextran can coat many nanoparticles to protect drugs that have a short half-life or can be easily decomposed. Taking advantage of this, Burova et al. used dextran as a hydrogel to protect insulin and obtained good results, which provides a result for the development of oral protein pharmaceutical preparations [50]. These studies prove that dextran has been used as a delivery vehicle for various drugs and is suitable for drug delivery.

Changing the drug release behavior through molecular self-assembly into hydrogels is another direction of dextran research. Similar to other hydrogels, dextran hydrogels can realize pH-responsive, redox-responsive, and sustained/controlled drug delivery. In 2018, Arvind K. Singh Chandel et al. used a partially long-chain alkylated dextran-graft-poly [(2-dimethylamino)ethyl methacrylate] copolymer to entrap hydrophobic guest drugs, after which reactive PEG was added to an aqueous solution to form an alkylated Dex-g-PDMA-linked-PEG co-network hydrogel. Compared with conventional hydrogels, this method can minimize the impact of organic solvents on pharmaceuticals, enhance mechanical properties and bioadhesive stress, and lower the degree of water swelling [51]. Although this new method has many advantages, conventional dextran hydrogels are also very innovative. For example, Wang et al. introduced an acid-labile hydrazone bond into redox-sensitive Dex-SS-PAA through one-step self-assembly so that pH- and redox-controlled drug release could be achieved [52].

### 3.3. Alginate

**Figure 6:**
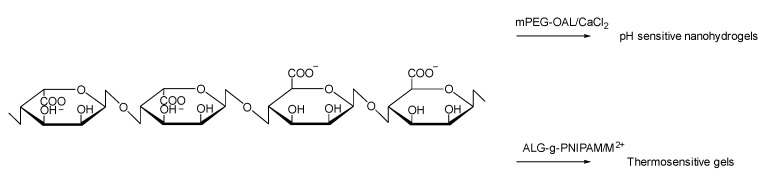
The structure of an alginate and its modification.

Alginate is a linear unbranched polysaccharide extracted from the cell walls of brown algae. This polymer is hydrophilic, and when it is hydrated, it can form a viscous gum [53,54]. There are two main processes to produce alginate: the alginic acid method and the calcium alginate method. The specific process is shown in the figure below. Compared with the first method, the disadvantage of the calcium alginate method is having an extra step in the process, and the advantage is that the operation of fibrous calcium alginate and alginic acid is simpler than the first one, and ethanol is not needed. It is composed of two 1–4 linked α-L-guluronate and β-D-mannuronate monomers, and the ratio of these two monosaccharides can change with different extraction sources (Figure 6). Alginate is an ocean-sourced polysaccharide, so it is a biopolymer and a polyelectrolyte, and has nontoxic, biocompatible, nonimmunogenic, and biodegradable characteristics; thus, it has been applied in many areas from an additive in the food industry to drug delivery [55,56,57,58]. Over the years, in the medical industry and pharmaceutics, alginates have been widely used for drug delivery and controlled release.

**Figure 7:**
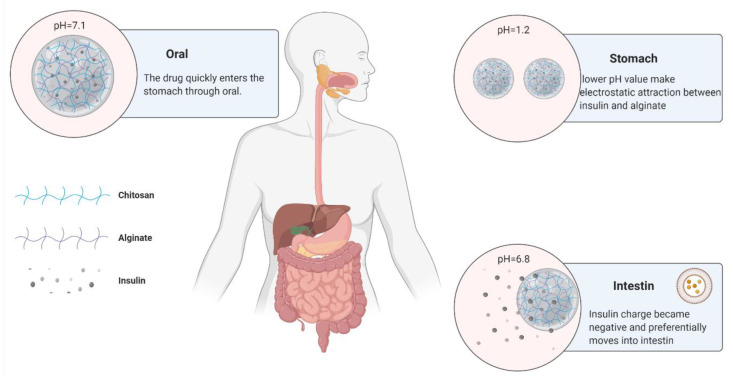
Use of chitosan and alginate to protect insulin.

Microspheres, gels, and nanoparticles are the three main applications of alginates in drug delivery systems. Alginates can form a gel with many divalent cations, for example, Ca^2+^, Zn^2+^ or Mg^2+^. This is easy to operate and very cheap for producing a bioactive microcapsule [59,60]. To use spray-drying techniques, extrusion and emulsification techniques, alginates can encapsulate proteins or other bioactive molecules to enhance absorption and molecular protection in different carrier systems. For example, in 2011, Zhang et al. used a membrane emulsification technique to chelate alginate-chitosan with Ca^2+^ and then solidify it to obtain insulin-loaded microspheres. This kind of insulin-loaded alginate-chitosan microsphere can be administered orally, as shown in Figure 7.

Animal experiments have shown that the glucose blood level in rats can be reduced and kept stable at a normal level for almost 60 h [61,62]. Similarly, this method can also be used as an encapsulation strategy for macromolecular proteins or bacteria [63,64].

Using alginate alone as a material to make sustained/controlled-release preparations is very rare now. To achieve a more precise drug release or better factor response, alginate needs to hybridize with other materials or iron, or be modified before use. For example, Peng et al. made hybrids of disulfide-modified alginate-based nanogels with superparamagnetic iron oxide nanoparticles to achieve dual responsive properties. This hybrid nanogel showed magnetic-targeted characteristics, loading large amounts of drugs, pH/redox dual-responsive properties, high toxicity to cancer cells, low toxicity to normal cells, and magnetic resonance imaging functions. Compared with early superparamagnetic iron oxide nanoparticles, this nanoparticle covered with alginate by disulfide bonds responded to two factors: the acidic microenvironment of tumor cells, and the high concentration of glutathione [39].

Alginate, as a vehicle for drugs, can combine with many different materials to enhance their ability in drug delivery, because of the characteristics of alginate—such as mechanical strength and porosity—are dependent on the ratios of guluronate and mannonate, type of ionic linker, and concentration of the solution. Cohen et al. demonstrated that alginate aqueous solution could become a gel in the eye without adding any ions. They adjusted pilocarpine release by changing the ratio of guluronic acid residues in the polymer backbone [65]. Thaned et al. used CG incorporated into SA and found that microenvironmental interactions between hydrated SA and calcium ions in distilled water could be created in formulations prepared to use a low compression force. Moreover, the incorporation of CG could moderate drug release and matrix erosion of the SA matrix capsules [66]. These results show that hybrid polymers and modified alginates are future direction, but alginate alone is not as important as that.

### 3.4. Gellan

**Figure 8:**
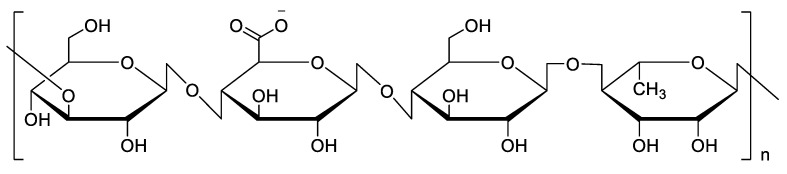
The structure of gellan.

Gellan is a water-soluble anionic carbohydrate polymer that was approved by the FDA in 1992; combined with the repeating unit, it consists of D-glucose, L-rhamnose, and D-glucuronic acid, as shown in Figure 8. There are low-acyl gellans and high-acyl gellans. Similar to their name, the difference between the two categories of polysaccharides depends on the number of acetate groups attached to the polysaccharide. The low-acyl polymer forms firm, nonelastic, brittle gels, whereas the high-acyl kind forms soft and flexible gels [67]. Therefore, researchers have studied the biological, chemical and physical characteristics of gellan, and explored its usage in nanopreparation and tissue engineering due to its unique features [68].

The most basic application of a gellan gun is as an adhesive, keeping the medication on the affected part of a patient’s body. For example, in 2016, Sneha et al. used gellan gum and cellulose to prepare a novel mucoadhesive in situ gel of salbutamol sulphate for nasal administration. In this study, gellan gum acted as a positively charged ion-sensitive carrier, allowing the drug to efficiently remain on the application site. The researcher used the gellan gun’s gelation rate and its proper viscosity to reduce mucociliary clearance, mucociliary transport, and the mucociliary escalator, as well as the first-pass effect and the doses for chronic diseases [69].

The use of gellan gum as the matrix of microparticles to manufacture sustained-release preparations is also one of its applications. Using a straightforward and cost-effective water-in-oil emulsion solvent diffusion method, Mukesh el al. prepared microcapsules that were compared with bare gellan gum microparticles, and the same microparticles but with methotrexate. The encapsulating efficiency and loading capacity of this dosage form are excellent with methotrexate. Fourier transform infrared spectroscopy confirmed that the GG polymer and methotrexate are very stable. Thermogravimetric analysis characterization showed that methotrexate-gellan gum MPs have a higher stability than gellan gum MPs. This study confirmed that methotrexate-loaded gellan gum microparticles suggested in vivo injectable ability in the future [70].

### 3.5. Pullulan

**Figure 9:**
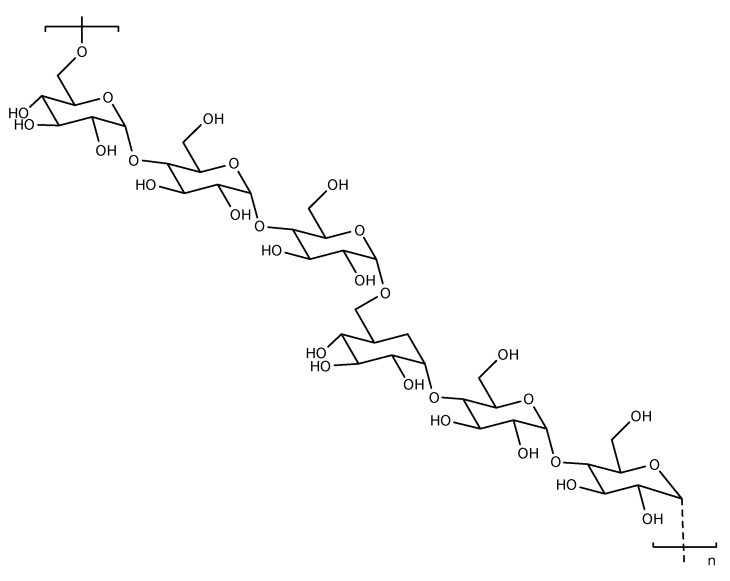
The structure of Pullulan.

Pullulan is a nonionic carbohydrate polymer produced from the fungus Aureobasidium pullulans. As shown in Figure 9, it is a neutral linear polycarbohydrate containing 1–6 linked maltotriose residues. It has a wide spectrum of characteristics, including biodegradability, thermal stability, and elasticity [71,72]. Pullulan itself is a highly water-soluble exopolysaccharide, but it is often modified to hydrophobized molecules and then applied in drug delivery systems, and different hydrophobizing strategies have been explored. Therefore, a variety of different drug delivery systems can be prepared by adjusting the hydrophilic and hydrophobic balance of pullulan, such as microparticles, nanoparticles, micelles, films and hydrogels.

The first application of pullulan was in microcapsules. In 2019, Yang et al. used the layer-by-layer self-assembly method to fabricate microcapsules combined with disulfide-crosslinked chitosan and hyaluronic acid. In this study, researchers loaded bovine serum albumin in microcapsules to research potential applications for protein delivery. They discovered that the disulfide bonds between chitosan and hyaluronic layers can remain stable for a long time, have low permeability under physiological conditions, and will degrade by cleavage when they are in a reducing environment. In addition to being redox-responsive, these microcapsules with good biocompatibility can be efficiently taken up by cancer cells via CD44-receptor-mediated endocytosis and release loaded proteins into cancer cells [73]. Changing the form of the drug to make it easier to administer is another function of pullulan. S.S. Santos et al. developed a clotrimazole-loaded eudragit nanocapsule for vulvovaginal candidiasis treatment, able to control antibiotic drug release and in vitro antifungal activity against Candida albicans. However, there are still some disadvantages of this dosage form because of drug leakage from the application site. After four years, a new dose form was developed based on Eudragit by the same research group. In 2017, Julia A. de Lima et al. designed a hydrogel containing pullulan as a final dosage form for clotrimazole-loaded Eudragit nanocapsules. This improvement showed that the nanocapsule suspension into a semisolid formulation was proper for vaginal administration. This dosage form is very promising as a substitute for the traditional drug of vulvovaginal candidiasis [74].

Nanoparticles are another main application of pullulan. In 2016, Ganeshkumar et al. used pullulan modified by acetate. This modified pullulan was adapted to curcumin nanoparticles to treat liver diseases. Curcumin has been used widely in recent years because some studies have proven that it can have anti-inflammatory, antioxidant, antibacterial, anticancer and cholesterol-reducing effects [75]. In recent years, amphiphilic copolymers and multistimuli-responsive polymers have been widely applied in drug delivery systems [76]. Therefore, in 2020, Xu et al. used a novel microwave-assisted method to synthesize amphiphilic poly(lactic-coglycolic acid)-graft-pullulan to prepare high-curcumin-loaded nanoparticles. These nanoparticles exhibited high temperature dependence in curcumin release. The next in vitro experiment elucidated that curcumin-loaded nanoparticles had on-demand drug release at high temperature and had a synergistic therapeutic effect on cancer [77].

### 3.6. Mannan

**Figure 10:**
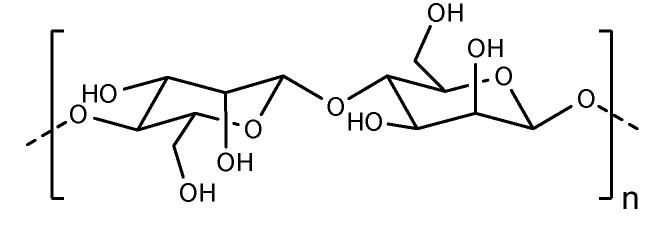
The structure of mannan.

Mannan is an important polysaccharide of the hemicellulose family, and it is a plant-storage carbohydrate consisting of mannose units, as shown in Figure 10. It occurs in a variety of plant tissues and in the cell walls of some seaweed. It is a highly branched polysaccharide that is categorized into four subfamilies: linear mannan, glucomannan, galactomannan, and galactoglucomanan. The structural characteristics of these polysaccharides is that the backbone consists of a β-1,4-linked mannose or a combination of glucose and mannose residues [78]. The most important roles of mannans are their structural, molecular, and storage functions. However, in 2007, Liepman et al. discovered that they are also a signal molecule in plant growth [79].

In comparison to other biocompatible natural polysaccharides, mannan-based drug delivery systems have additional properties, for example, easy recognition by mannose receptors expressed on the membrane of antigen-presenting cells, which promotes the uptake of mannose-bearing particles via mannose-mediated endocytosis and phagocytosis. Therefore, in 2019, Eliška et al. used a click reaction to link low-molecular-weight mannan with lipids to prepare functionalized liposomes. These functionalized nanoliposomes preserved mannan’s ability to deliver antigens or antimicrobials to dendritic cells and led to the activation of human dendritic cells, as detected by the expression of activation markers. This suggests that nanoparticles can be combined with protein-based, DNA-based or RNA-based vaccines to induce precision and long-lasting immune responses [80].

In addition to being a coat of nanoparticles to induce phagocytosis, mannan has also been applied to saturate the mononuclear phagocyte system. In 2020, Zakia et al. used the same characteristics of mannan to achieve mononuclear phagocyte system escape and efficient nanoparticle drug delivery. Cationized mannan-modified extracellular vesicles can help CD47-enriched exosome nanocarriers escape the endocytosis of macrophages, enhancing the tumor cell uptake of antitumor drugs. Experiments in mice demonstrated that the drug delivery system can induce a 123.53% increase in antitumor drugs in cancer cells compared with traditional nanocarriers [81]. These studies provide novel ideas for mannan applications in drug delivery systems.

### 3.7. Hyaluronan

Hyaluronan, or hyaluronic acid (HA), is one of the largest elements of the extracellular matrix and is the only glycosaminoglycan produced on the cell membrane. It is a polysaccharide made up of the disaccharide formed by D-glucuronic acid and D-N-acetyl glucosamine linked by alternating 1–4 and 1–3 glycosidic bonds. As another polysaccharide, hyaluronan also shows excellent biocompatibility and biodegradability. However, hyaluronan also has its own characteristics, such as mechanical and structural characteristics in the synovial fluid or the vitreous humor, and sometimes it interacts with cells to trigger some responses. After being modified, hyaluronan can self-organize in aqueous solution; therefore, it can be viewed as a suitable vehicle for drug delivery.

The earliest drug delivery system based on HA was the ophthalmic hydrogel, which plays an essential role in tissue hydration and lubrication. Based on experiments in humans and rabbits, they found that with hyaluronic acid gel, the precorneal clearance of pilocarpine was slower than that of its solution, and the precorneal residence time could be prolonged. This means that HA can therefore be used as a suitable vehicle for controlled drug delivery to the eye [82]. After that, an increasing number of kinds of drug carrier platforms have been developed, such as nanogels, nanoparticles, nano capsules, and hydrogels.

As a bioactive molecule, HA can have many different functions by changing its size or concentration [83,84]. It can protect creatures from aging and from tumor development. It is very interesting that HA fragments have a totally different effect than intact molecules with high molecular weights. Combining HA with CD44 is one of the most important uses of HA in drug delivery systems because of the high level of CD44 expression on cancer cells and inflammatory macrophages. As an active targeting polymer, HA is widely used in cancer-targeting carriers, and HA not only exerts its targeting effect but also uses its physical properties to achieve the combination of targeting CD44 and pH-responsive, reactive oxygen species (ROS)-responsive or photothermal- and photodynamic-sensitive materials, for both in vivo photothermal imaging and drug delivery [76].

ROS are a very important class of molecules involved in biological events ranging from cell homeostasis to proliferation to cell death, and they are also expressed at very low levels in the normal organ microenvironment. Nevertheless, many diseases, such as cancer and inflammation, lead to the localized overexpression of ROS. Therefore, this class can be designed as a target for the treatment of some diseases. In 2008, Hyukjin Lee et al. used gold nanoparticles functionalized with end-immobilizing near-infrared fluorescence dye-labeled hyaluronic acid for the in vivo-detection of some diseases, such as lung cancer or diseases that have an inflammatory response, which can result in the overexpression of ROS. Initially, the fluorescence from dye conjugated on the nanoparticle quenches near the metal surface because of the nanoparticle surface energy transfer; after the linker between the fluorescent dye and HA is degraded because of ROS, the fluorescence recovers. As the ROS level increases, the fluorescence of this nanoparticle increases, showing the dose-dependent detection ability of this nanoformulation. After in vivo experiments, the nanoparticles preferentially accumulated in tumors via the CD44 receptor interaction of HA, followed by the recovery of the fluorescent signals [85].

**Figure 11:**
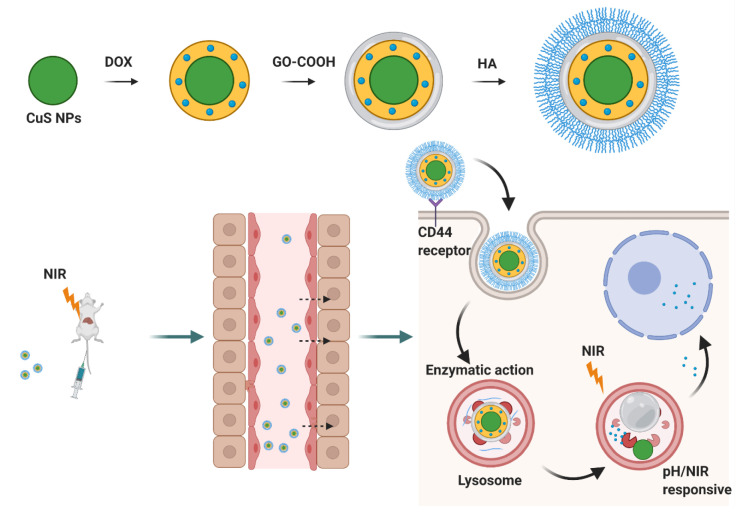
NIR and pH responses of hyaluronic acid nanogels.

In addition to ROS, the combination of photodynamics or photothermal therapy for targeted drug delivery has recently become a very popular research direction for HA. In 2020, Kishwor Poudel et al. used dual photothermal agents, including copper sulfide and graphene oxide, doxorubicin, and HA, to merge into a complexed nanoparticle for triple-responsive therapy. As indicated in Figure 11, due to HA’s targeting of CD44 and its characteristics, the nanodelivery system can target CD44-overexpressing cells and avoid unexpected release; at the same time, it can target cancer cells. Simultaneously, hyperthermia, photodynamic effects and high ROS levels were observed in the presence of dual photosensitizers CuS and GO [86].

Similar to other polymer carbohydrates, hyaluronic acid can be used to make hydrogels. Fu et al. developed a gelation prodrug for the sustained release of doxorubicin hydrochloride (DOX·HCl). To achieve this sustained release effect, DOX·HCl was chemically conjugated onto thiolate HA by an acid-sensitive hydrazone bond. When those molecules are exposed to air, they begin to conjugate to the hydrogel. The number of thiol groups can adjust the gelation time and extent on HA. The pH and reductant are two important factors of the hydrogel-released conjugated DOX. HCl and in vitro experiments confirmed the efficacy and targeting of these hydrogels for cancer cell inhibition [87].

### 3.8. Chitin, Chitosan, and Chitosan Oligosaccharides

Chitin is the second most abundant natural biodegradable polymer after cellobiose. It is obtained from many different sources, including carapaces of marine creatures, insect skeletons, and the exoskeletons of invertebrates. The structure of chitin is defined as a β-(1–4)-linked linear cationic heteropolymer consisting of 2-acetamide-2-deoxy-D-glucopyranose and randomly distributed units of 2-amino-2-deoxy-D-glucopyranose. The degree of chitin acetylation exceeds 90%, so innumerable intermolecular and intramolecular H-bonds can form between them [88]. Due to its biodegradability, biocompatibility, nontoxicity, physiological inertness and gel-forming properties, chitin has a wide range of applications in drug delivery [89]. As the deacetylation product of chitin, chitosan is the only natural cationic polysaccharide known [90]. COSs are depolymerized products of chitosan [91].

Chitin, chitosan, COS and their derivatives have extensive use in drug delivery systems. Because of their characteristics, such as biocompatibility, biodegradability, and low toxicity, they are highly important polymers that can encapsulate active pharmaceutical ingredients to protect them against decomposition and extend drug release [92]. Similar to other carbohydrate polymers, chitin, chitosan, and COS can be made into different drug delivery systems, such as nanoparticles, microcapsules, or hydrogels.

In recent years, the novel usage of chitin in the field of drug delivery has been nanocrystalline particles. In 2018, Drozd et al. published an article confirming that nanoparticles constructed by chitin have no platelet aggregation or secretion when their concentration is below 0.63 mg/mL [93]. This result proved that nanocrystalline chitin has the potential to be a drug delivery system. However, most research programs on nanocrystalline chitin have concentrated on its manufacturing method and mechanical properties [94], but there is still very little research on its drug loading [95]. Chitin-based core–shell microspheres are another pharmaceutical type for chitin drug delivery. The biological properties of these microspheres and their dual-drug release behaviors were studied by Y. Shang et al., 2018 [96]. They used coaxial electrostatic liquid droplets to produce acrylamide-modified chitin or chitosan core–shell-structured microspheres. The author proved the structure and morphology of these microspheres by FT-IR and SEM. The release behaviors of blue dye suggest that low molecular weight drugs are released faster than high molecular weight drugs, so they can be designed for diseases that need different drugs and different drug release durations. 

Chitosan has many features, such as biodegradability, biocompatibility, hydrophilicity, nontoxicity, high bioavailability, sample to modification, selective permeability of water, and excellent chemical resistance, so it can form films, gels [97,98], nanoparticles, microparticles, and beads. Additionally, because chitosan is biodegradable, it can decompose in vivo to amino sugars, which can be easily absorbed by the human body. Therefore, chitosan and its derivatives have been broadly researched in many drug carriers [99]. In a recent study, Seila Tolentino et al. used chitosan and hyaluronic acid to produce nanoparticles that entrap clindamycin. Simultaneously, they evaluated the effect of these two nanoparticles on precision drug delivery to pilosebaceous units for the first time. Chitosan and hyaluronic acid nanoparticles had opposite surface charges (+27.7 ± 0.9 mV and −30.2 ± 2.7 mV, respectively). Compared with the commercial formulation, both kinds of nanoparticles showed enhanced target ability. Remarkably, there is potential targeting ability when the skin is pretreated to simulate a sebaceous condition [100]. In 2019, Liang et al. used chitosan-grafted dihydrocaffeic acid and oxidized pullulan to construct an injectable pH-sensitive hydrogel. This carrier system has excellent mucoadhesiveness, so it can be applied to colon cancer drug delivery or mucoadhesive drug delivery. To solve the problem of chitosan’s poor solubility and obtain the adsorption ability of mucosa, they grafted dihydrocaffeic acid to the 2-amino of D-glucopyranose. After that, pullulan was mixed with this chitosan-grafted dihydrocaffeic acid to obtain the hydrogel. In subsequent in vitro and in vivo experiments, this drug delivery system was proven to have good pH responsiveness when pH = 5.5, and hydrogel formed in ten minutes and adhered at the injection position on the rat skin [101,102]. Chitosan also has the advantage that it can interrupt cellular tight junctions, and this characteristic endows it with the ability to cross the blood–brain barrier (BBB) [103,104]. Therefore, it has been complexed with n-butylglycidyl ether to form nanoparticles that can be used as a drug carrier system that can cross the BBB. To date, chitosan nanoparticles have been used with bromocriptine [105], levodopa [106], pramipexole [107], selegeline [107], rotigotine [108] and ropinirole [109] to improve their ability to cross the blood–brain barrier. Chitosan can also be used as the outer shell of metal nanoparticles. In the study of Gounden et al., AgNPs were synthesized, functionalized with CS, and loaded with the anticancer drug cisplatin (CIS). The encapsulated drug was rapidly released from the AG-CS-CIS complex at low pH, favoring delivery to the tumor microenvironment [110].

One of the early applications of COS in drug delivery systems was by Mac Laughlin et al., who prepared a delivery system for transferring DNA plasmids. The results showed that compared with naked plasmids, plasmids with COS could be expressed, demonstrating that COS can be effectively applied to transfer plasmids in vivo [111]. COS can also be modified so that it can be used by different drug carriers. For example, Hu et al. developed a stearic acid-grafted chitosan carbohydrate vehicle to encapsulate DOX. These DOX-conjugated stearic acid-g-chitosan oligosaccharide polymeric micelles (DOX–CSO–SA) indicated pH-dependent DOX release behavior. When the pH for the release medium was reduced from 7.2 to 5.0, the release rate of DOX from DOX–CSO–SA micelles showed a great increase. The in vivo anticancer cell activity results showed that DOX–CSO–SA treatments effectively reduced tumor growth and that the toxicity of DOX to the animal body was lower than that of the existing doxorubicin hydrochloride injection [112].

### 3.9. Agarose

**Figure 12:**
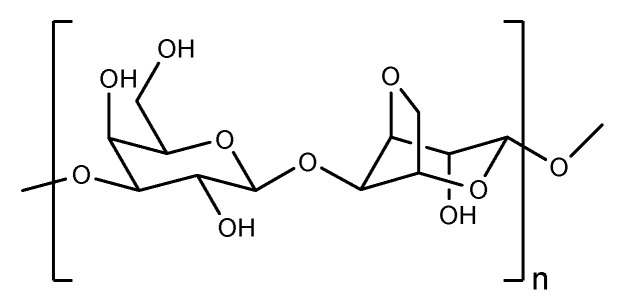
The structure of agarose.

Similar to chitosan, agarose, as shown in Figure 12, is another prominent marine polysaccharide that has good mechanical properties, high bioactivity, and switchable chemical reactivity for functionalization. However, compared with chitosan, it has its own characteristics, such as solubility in neutral or alkaline environments and neutral surface charge stability in different pH environments. Therefore, it has many distinctive application directions.

In 2015, Mak et al. took advantage of agarose’s neutral surface charge stability to develop temperature-responsive microcapsules for the delivery and subsequent controlled release of cells. Because agarose does not provide attachment points, these can be provided by naturally derived extracellular matrix proteins, so cells will not be lost by anoikis [113]. The formation of amphiphilic co-network (APCN) gels is another important application of agarose. In 2015, Anupam Bera et al. used modified agarose to prepare different APCN gels. These APCN gels exhibited a cocontinuous nanophase morphology, pH-responsive water swelling, and the pH-triggered release of hydrophobic and hydrophilic drugs. These gels are biodegradable, so the milled APCN gel particles are theoretically safe enough to inject through a hypodermic syringe [114].

### 3.10. Guar Gum

**Figure 13:**
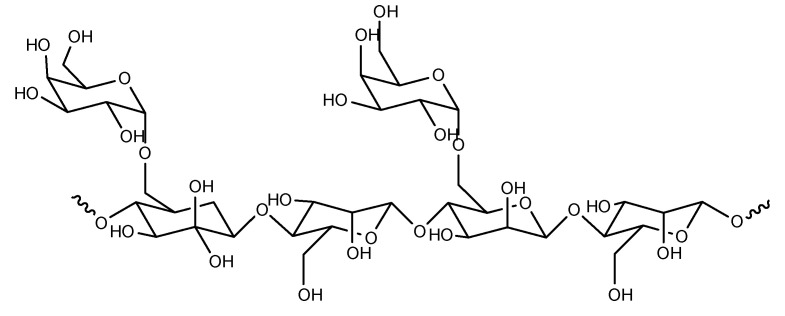
The structure of guar gum.

Guar gum (Figure 13) is a natural polysaccharide with the characteristics of easy availability, low cost, excellent viscoelastic properties and nontoxic properties. However, as a drug delivery carrier, the usage of guar gum is limited because of its high swelling characteristics in aqueous media. A representative study by Amna Farooq et al. in 2022 used silane as a crosslinker to prepare pH-sensitive hydrogels [115]. They used vinyltriethoxy silane (VTEOS) to optimize Kappa-carrageenan (KC)/guar gum (GG)/poly(vinyl alcohol) (PVA)’s high swelling characteristics. In this study, the increase in the concentration of VTEOS and the swelling ratio decreased in water, whereas it showed maximum swelling in acidic media, low swelling in basic media and moderate swelling at neutral pH. This research will lead to the wider application of guar gum in the field of pharmaceutical formulations.

### 3.11. Carbohydrate-Based Materials in Phase

Even though carbohydrate-based materials are widely used in the laboratory as scaffolds, their application in drugs that are in phase is very limited. The first drug-applied carbohydrate-based material was a scaffold named GXV (code name), which became available on Mar 20, 2001. This was a DNA vaccine formulated in chitosan nanospheres. However, the highest phase of this drug was preclinical, and it was not eventually entered into the clinic. After 4 years, CHP-HER2 was disclosed as a drug in cancer immunotherapy. It was the first drug to enter clinical trials (ClinicalTrials.gov Identifier: NCT00291473) that used carbohydrate-based materials as scaffolds. This is a protein antigen delivery system consisting of cholesteryl pullulan (CHP) nanogels complexed with soluble protein molecules in an attempt to present peptides that can bind both MHC class I and class II molecules in APCs. Except for this drug, a total of four drugs entered the clinic, including PRV-111, IMF-001, CHP-NY-and ESO-1, as shown in Table 1

In addition to the drugs for the treatment of disorders, reagents for diagnostics that use carbohydrate-based materials are developing very quickly in the clinic. In 1994, Bayer disclosed dextrans agnetic resonance imaging (MRI) contrast-agent nanoparticles named Cliavist Resovist that could diagnose cancer and liver diseases, and this reagent was launched in 2001. In the same year, AMAG Pharmaceuticals disclosed Combidex Sinerem, a very similar reagent to Cliavist Resovist. It is a superparamagnetic dextran T-10-coated iron oxide colloid with particles that range from 10 to 20 nm in diameter, with an apparent mass of 800,000 daltons.

In surgery, carbohydrate-based materials are also widely used. In 2021, Arvind K. Singh Chandel et al. put together a series of examples of the use of carbohydrate polymers in postoperative peritoneal adhesion. From this review, alginate, hyaluronan, cellulose, starch, chondroitin sulfate, and polyethylene glycol showed very good properties compared to conventional antiadhesion barriers. The conclusion of the limitations on current antiadhesion barriers identified the necessary direction for the subsequent development of antiadhesion barriers [116].

Although very few drugs have entered clinical testing, the drugs that have moved into preclinical and biological testing are large. To date, 178 drugs that use carbohydrate-based materials as scaffolds have been disclosed for preclinical biological testing and clinical testing. Since 2012, the number of drugs has gradually increased to show a bright future for this kind of pharmaceutical.

## 4. Carbohydrate-Modified Materials for Targeted Drug Delivery Systems

Carbohydrates, as a compound of living organism energy and an information molecule in vivo, play an important role in physiological processes and can control the communication of in vivo information by recognition with biological molecules. As a specific recognition biological molecule, some sugars targeting special receptors can be used as drug molecules or drug carriers, which is called glycosylation modification. Three kinds of carbohydrates can be used in this glycosylation modification drug delivery system: mannose [117]; galactosyl [118]; and glucosyl [119].

**Figure 14:**
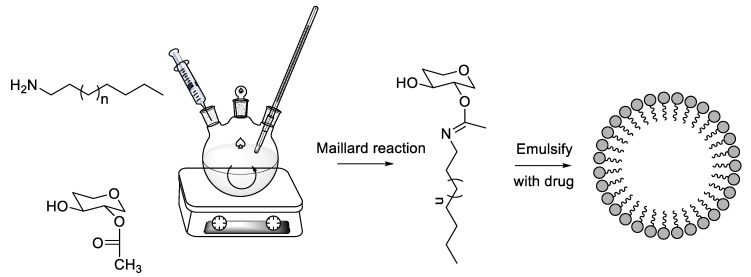
Application of the Maillard reaction in carbohydrate-modified preparations.

As one of the most likely target carbohydrates, mannose is often applied in the modification of anticancer medicine and the drug delivery systems related to this. To use the Maillard reaction, as shown in Figure 14, mannose can link with many drug carriers that have amino groups, such as proteins and amino-rich liposomes. The C-type lectin receptor and GLUT1 are the main binding sites. In 2017, Kim et al. synthesized an amino-polyethylene glycol-polylactic acid-glycolic acid complex and then prepared the complex with nanoparticles modified by amino groups. At the same time, they used a reductive amination reaction to modify the nanoparticles with mannose to target macrophages. These nanoparticles were loaded with diclofenac and then coated on sutures. Diclofenac is a nonsteroidal anti-inflammatory drug that removes pain and swelling. After in vivo and in vitro experiments, the results showed that the anti-inflammatory effect was enhanced in both macrophage and excisional wound healing animal models compared to the free drug molecule-coated suture. Compared with traditional sutures, sutures with those nanoparticles vastly depressed prostaglandin E2 synthesis of Raw264.7 cells, compared to free diclofenac, and reduced the inflammatory reactions of tissues surrounding the suture in an animal model [120]. This study showed that mannose has a huge potential in reducing in vivo inflammation and pain at the site of injury.

Glucosyl can also couple with polymers by the Maillard reaction. Li et al. first prepared glucose-connected chitosan nanoparticles for specific recognition and binding with glucose transporters overexpressed by cancer cells. They linked glucosamine and chitosan by using succinic acid. After entrapment with doxorubicin, the nanoparticles with glucosyl showed endocytosis ability greater than chitosan nanoparticles without it, and the antitumor activity of this drug delivery system was 4–5 times more effective in 4T1 cell killing than chitosan nanoparticle doxorubicin without glucosyl. The results showed that glucose-bound chitosan nanoparticles could be a drug carrier that target cancer cells, because these cells have many glucose transporters on the surface. Carbohydrates can also be combined with novel polymers, such as polyvinyl-grafted caprolactam-polyvinyl acetate-polyethylene glycol, which is a new amphiphilic nonionic medical polymer material [121]. Moretton et al. also used glucose as a modification group to prepare paclitaxel micelles targeting breast cancer. The results confirmed that glucose-modified nanoparticles loaded with paclitaxel actually have the potential to treat breast cancer [122].

As a liver-targeting modification group [123], galactose, which is very popular in the recent RNA therapy field, is also a commonly used targeting agent modification group. Zou et al. prepared paclitaxel micelles that use different numbers of galactoses for modification. With the increase in galactose on PEG, the half-inhibitory concentration of the drug micelles on cancer cells steadily declined. Simultaneously, with covalent crosslinking of the micellar core, paclitaxel micelles have very good stability with inhibited drug leakage under physiological conditions. However, when this carrier was at pH 5.0, the release of paclitaxel was significantly enhanced compared with when it was at pH 7.0. This study proved that galactose provides an interesting platform for targeted cancer and has great potential when combined with other drug delivery platforms [124]. Although there are currently few chemical methods to connect RNA or DNA with carbohydrates, an increasing number of novel methods have been developed by scientists [125,126,127].

However, there are some limitations and problems in the application of glycosylation in the synthesis of a precise drug carrier system. For instance, there is an unmistakable connection between different types of glycosylation and the number of glycosylation gatherings, as seen by the performance of the medication conveyance framework. The ideal glycosylation forms and the quantity of glycosylation bundles shift as indicated by various sickness models. Currently, the glycosyl ligands utilized for glycosylation adjustment are generally single carbohydrates; however, there are few investigations on polycarbohydrates. Additionally, not every medication conveyance framework is appropriate for glycosylation adjustment. Some medication conveyance frameworks may bring about decreased adequacy after glycosylation alteration. Furthermore, glycosylation-adjusted medication conveyance frameworks may focus on different organs with glycosyl receptors. Nonetheless, there are currently few examinations that focus of various tissues. The majority of these glycosylation-focused drug conveyance frameworks are assessed by in vitro trials, and more in vivo explorations are expected to further check their safety.

## 5. Conclusions

Carbohydrates, as one of the most abundant organic compounds in nature, have broad-spectrum chemical structures and biological functions, and they have a long history in drug delivery systems. Because they are a polymer that has nontoxicity, biocompatibility, and biodegradability, they play an important role in drug delivery applications. These excipients, nanoparticles, nanocomposites and hydrogels made by carbohydrates can help control the speed of the drug, increase the targeting of drugs, protect the structural integrity of the drug, and be sensitive to the microenvironment. After many years, drug delivery systems that simply use carbohydrates have developed and improved, and are now achieving maturity.

Currently, biotherapy DNA/RNA and proteins are playing an increasingly important role in the field of disease treatment. Thereby, the future direction of macromolecular polysaccharide preparations should not only be limited to the field of small molecule drugs, but should also be increasingly directed towards improving the delivery of cells, for example, DNA/RNA and proteins, preventing them from being degraded in the body, effectively delivering them to the inside of cells, and improving their oral bioavailability. Modified carbohydrates and complexes with other materials are future research directions in this area.

## Figures and Tables

**Table 1:** The drugs used incorporating carbohydrate-based materials as scaffolds entering clinical trials.

Code Name	Drug Name	Available Date	Highest Phase	Prescription/Indication Type	Condition	Organization	Related Basic Patent
PRV-111	Cisplatin ChemoThin Wafer (Generic) PRV-111 (Code)	6 November 2015	Phase I/II	Orphan drug designation	Cancer, anus cancer, head and neck cancer, mouth (squamous cell) cancer, salivary glands	Privo Technologies University of Massachusetts	US 2014234212
IMF-001	IMF-001 (Code)	31 December 2010	Phase I	Nanoparticles of cholesteryl hydrophobized pullulan	Cancer, esophagus cancer, solid tumor	ImmunoFrontier	
CHP-NY-ESO-1	CHP-NY-ESO-1 (Code)	10 October 2006	Phase I	Nanoparticles consist of cholesterol-bearing hydrophobized pullulan	Cancer, solid tumor	Ludwig Institute for Cancer Research	
CHP-HER2	Cholesteryl hydrophobized polysaccharide-Her2 protein complex (Generic)CHP-HER2 (Code)	3 November 2005	Phase I	Nanoparticles consist of cholesterol-bearing hydrophobized pullulan	Cancer	Mie University Nagasaki University	
SHU-555AZK-132281	Ferucarbotran (Generic) Magnetites (Generic) Cliavist (Brand) Resovist (Brand) SHU-555A (Code) ZK-132281 (Code)	25 November 1994	Launched 2001	Superparamagnetic iron oxide covered with Dextrans	Cancer, liver diagnostics liver diseases	Bayer Epix Pharmaceuticals Freie Universitaet Berlin (FU Berlin) Meito Sangyo Nihon Schering Schering AG	WO 2006028129EP 2005970WO 2010049062US 2012095325WO 2012136813WO 2013177364
[68Ga]Nanocolloid	[68Ga]Nanocolloid (Code)	24 January 2019	Clinical	Nanoparticles consist of Dextrans and gallium complexes	Cancer, prostate	Peter MacCallum Cancer Centre (Originator)University of Melbourne (Originator)	
